# Editorial: Insights in rehabilitation for musculoskeletal conditions 2023/2024

**DOI:** 10.3389/fresc.2025.1729389

**Published:** 2025-11-26

**Authors:** Maciej Płaszewski, Josette Bettany-Saltikov

**Affiliations:** 1 Faculty in Biała Podlaska, Józef Piłsudski University of Physical Education in Warsaw, Warsaw, Poland; 2 Teesside University, Middlesbrough, United Kingdom

**Keywords:** rehabilitation, functioning, musculoskeletal, measurement, lived experiences, terminology, physical activity

The past decade has witnessed a profound evolution in the understanding of *rehabilitation for musculoskeletal conditions* – from a narrow focus on impairment reduction to a broader, human-centered orientation toward functioning [which was also experienced by the editors of this Research Topic (1–3)]. The Rehabilitation is increasingly recognized not as a downstream intervention but as a key health strategy enabling individuals to live well, not merely live longer (4). This shift aligns with what Cieza (5) termed the “*functioning revolution”*—a call for rehabilitation stakeholders to unify diverse approaches under the common goal of optimizing functioning as both outcome and process (6).

## Functioning as the third health indicator

From a public health perspective, functioning has emerged as the third health indicator, complementing morbidity and mortality (7). Beyond quantifying disease or death, it reveals *how* people live – their ability to act, participate, and engage within their environments. This conceptual expansion demands that rehabilitation science addresses not only body structures and impairments, but also activity, participation, and contextual factors, as framed by the International Classification of Functioning, Disability and Health (ICF) (8).

Across this Research Topic, the contributing authors illuminate how assessment, technology, culture, and system design are converging to realize this vision. Together, their work underscores that the future of rehabilitation for musculoskeletal conditions depends on the integration of biomedical, psychosocial, and digital paradigms, as well as on equitable access and interdisciplinary collaboration.

## Global burden and the imperative of equity

Musculoskeletal disorders are the most prevalent health conditions that could be addressed through rehabilitation. Low back pain, the most common musculoskeletal disorder, is the leading cause of years lived with disability (9, 10). Liu et al. *[this Research Topic]* provide a global perspective, showing that the burden of musculoskeletal disorders (MSDs) will continue to rise through 2050, driven by demographic aging and unequal healthcare access. Their analysis highlights a pressing need for strategically targeted interventions and resource redistribution to mitigate disability and premature retirement linked to MSDs. The call for tailored approaches across both high- and low-SDI countries resonates with the global rehabilitation agenda's emphasis on equity, inclusion, and sustainability – principles central to strengthening rehabilitation within health systems.

## Functioning-centered innovation in practice

Rehabilitation evolves through innovation, and several contributions in this collection demonstrate how new technologies and integrative approaches can expand the scope of functioning-oriented care.

Liguori et al. *[this Research Topic]* present an inspiring vision of digital empowerment in rehabilitation: a mobile application designed to improve exercise adherence and self-management in patients with knee osteoarthritis. By bridging clinical expertise and patient self-efficacy, their work exemplifies how technology can foster personalized and cost-effective rehabilitation, supporting functioning in everyday environments.

In a different yet complementary direction, Wang and Bao *[this Research Topic]* show the effectiveness of integrating traditional Chinese bone setting with neuromuscular electrical stimulation (NMES) in office workers with lumbar disc herniation. Their results highlight that combining traditional knowledge with evidence-based modern techniques can yield faster recovery, improved lumbar stability, and lower recurrence – demonstrating how cultural inclusiveness and innovation can coexist within evidence-based rehabilitation.

## Language, collaboration, and conceptual unity

Rehabilitation science continues to face conceptual challenges—among them, the need for terminological and interdisciplinary coherence. *Brindissino*, Turgut, and Struyf *[this Research Topic]* provide a critical reflection on frozen shoulder terminology and classification, showing how inconsistent use of terms can hinder both research and clinical communication. Their proposal for unified terminology represents a step toward shared understanding and improved integration of findings across contexts.

Similarly, Macrelli et al. *[this Research Topic]* highlight gaps in collaboration between physiotherapists and orthopaedic surgeons in the management of anterior cruciate ligament (ACL) injuries. Their findings emphasize the importance of mutual knowledge exchange and the integration of non-surgical rehabilitation strategies into mainstream care pathways. This collaborative spirit embodies the biopsychosocial model in action – where expertise converges around a shared goal of optimizing functioning.

## Assessment, measurement, and the lived experience of functioning

Assessment remains a cornerstone of rehabilitation, yet as Zidan et al. demonstrate in their work on arthrogryposis multiplex congenita (AMC), existing functional mobility measures often fail to capture the full lived experience of functioning. Their analysis of instruments such as FMS, FAQ, WeeFIM, and PROMIS underscores the need to include environmental challenges, compensatory strategies, and pain in functional outcome assessment.

At the same time, Stofberg et al. *[this Research Topic]* contribute valuable methodological insights into monitoring rehabilitation progress after ACL reconstruction. Their findings show that isometric mid-thigh pull peak force (IMTP PF) can sensitively track changes in force capacity and asymmetry, revealing that both the injured and uninjured limbs adapt positively to rehabilitation. Importantly, their study highlights that ACL reconstruction, though a unilateral injury, requires bilateral rehabilitation—a reminder that functioning is systemic, not localized.

## Looking forward: toward a functioning-centred rehabilitation ecosystem

Taken together, the studies in this Research Topic illustrate a field that is simultaneously expanding and converging – expanding through technological and methodological innovation, with additional focus on terminology refinement and clarification. The next decade of musculoskeletal rehabilitation will demand:

- deeper integration of digital tools, data-driven insights, and patient-generated outcomes;
- system-level reforms ensuring equitable access and interprofessional collaboration;
- and continued development of functioning-based outcome frameworks that capture the diversity of human experience.

As Krug and Cieza (4) wrote, strengthening rehabilitation services will enable millions not only to *live longer* but to *live well*. The contributions collected here show that this aspiration can be realized – through science, through collaboration, and through a shared global commitment to functioning as both compass and measure of progress.
